# Propulsive Power in Cross-Country Skiing: Application and Limitations of a Novel Wearable Sensor-Based Method During Roller Skiing

**DOI:** 10.3389/fphys.2018.01631

**Published:** 2018-11-20

**Authors:** Øyvind Gløersen, Thomas Losnegard, Anders Malthe-Sørenssen, Dag Kristian Dysthe, Matthias Gilgien

**Affiliations:** ^1^Condensed Matter Physics, Department of Physics, University of Oslo, Oslo, Norway; ^2^Department of Physical Performance, Norwegian School of Sports Sciences, Oslo, Norway; ^3^Department of Physics, Center for Computing in Science Education, University of Oslo, Oslo, Norway; ^4^Department of Sport Science, Alpine Skiing, Norwegian Ski Federation, Oslo, Norway

**Keywords:** work rate, force, energy, GNSS, GPS, validity

## Abstract

Cross-country skiing is an endurance sport that requires extremely high maximal aerobic power. Due to downhill sections where the athletes can recover, skiers must also have the ability to perform repeated efforts where metabolic power substantially exceeds maximal aerobic power. Since the duration of these supra-aerobic efforts is often in the order of seconds, heart rate, and pulmonary VO_2_ do not adequately reflect instantaneous metabolic power. Propulsive power (*P*_prop_) is an alternative parameter that can be used to estimate metabolic power, but the validity of such calculations during cross-country skiing has rarely been addressed. The aim of this study was therefore twofold: to develop a procedure using small non-intrusive sensors attached to the athlete for estimating *P*_prop_ during roller-skiing and to evaluate its limits; and (2) to utilize this procedure to determine the *P*_prop_ generated by high-level skiers during a simulated distance race. Eight elite male cross-country skiers simulated a 15 km individual distance race on roller skis using ski skating techniques on a course (13.5 km) similar to World Cup skiing courses. *P*_prop_ was calculated using a combination of standalone and differential GNSS measurements and inertial measurement units. The method's measurement error was assessed using a Monte Carlo simulation, sampling from the most relevant sources of error. *P*_prop_ decreased approximately linearly with skiing speed and acceleration, and was approximated by the equation $P_{\text{prop}}(v,\dot{v}$) = −0.54·*v* −0.71$\cdot \dot{v}$ + 7.26 W·kg^−1^. *P*_prop_ was typically zero for skiing speeds >9 m·s^−1^, because the athletes transitioned to the tuck position. Peak *P*_prop_ was 8.35 ± 0.63 W·kg^−1^ and was typically attained during the final lap in the last major ascent, while average *P*_prop_ throughout the race was 3.35 ± 0.23 W·kg^−1^. The measurement error of *P*_prop_ increased with skiing speed, from 0.09 W·kg^−1^ at 2.0 m·s^−1^ to 0.58 W·kg^−1^ at 9.0 m·s^−1^. In summary, this study is the first to provide continuous measurements of *P*_prop_ for distance skiing, as well as the first to quantify the measurement error during roller skiing using the power balance principle. Therefore, these results provide novel insight into the pacing strategies employed by high-level skiers.

## Introduction

Current cross-country ski races can be categorized into two main events; sprint skiing (<1.8 km) and distance skiing (>10 and 15 km, female and males respectively). Furthermore, races can be held using free technique, where athletes typically choose to use ski skating techniques, or as classic technique races, where skiers are restricted to specific sub-techniques (herringbone, diagonal stride, double poling, kick double poling). Due to these restrictions, the average race speed in free technique events is typically about 10% higher than in classical technique races (Bolger et al., 2015). Regardless of events, the race course regulations specify that courses should contain approximately equal parts of uphill, downhill, and flat terrain, to “test the skier in a technical, tactical and physical manner” (FIS Cross-Country Homologation Manual, June 2017).

Regardless of race distance, cross-country skiing is an endurance sport that demands an exceptionally high aerobic energy turnover, in addition to high movement efficacy. This is underlined by the fact that elite cross-country skiers have among the highest maximal oxygen consumptions of any sports (Sandbakk and Holmberg, 2014; Haugen et al., 2017), typically ranging from 80 to 90 and 70 to 80 mL·kg^−1^·min^−1^ for world class males and females, respectively (Ingjer, 1991; Losnegard and Hallén, 2014; Sandbakk et al., 2016a). In addition, several studies also indicate substantial anaerobic turnover rates during a race, a phenomenon attributed to the large variations in course inclination. Moreover, skiers typically choose to increase their metabolic power in uphill terrain (Karlsson et al., 2018), often attaining a metabolic power that substantially exceeds their peak aerobic power (estimated at 110–160% of VO_2peak_ Norman and Komi, 1987; Sandbakk et al., 2011; Karlsson et al., 2018). These repeated supra-aerobic efforts vary in duration from seconds to minutes, and incur an oxygen debt that must be recovered in the downhill or flat sections (Sandbakk and Holmberg, 2014; Karlsson et al., 2018). Such transient changes in energetic demand are not well reflected by measurements of pulmonary VO_2_ or heart rate, because both have a blunted response due to the use of local oxygen stores and anaerobic energy pathways. Hence, both parameters behave as if they passed through a lagged low pass filter and remain high (85–95% of their peak values) throughout the race (Welde et al., 2003; Bolger et al., 2015; Karlsson et al., 2018).

The combination of high and sustained aerobic energy turnover with repeated supra-aerobic efforts distinguishes cross-country skiing from many other endurance racing sports, where the work rate is relatively constant and requires measurement of parameters that reflect the instantaneous energy demands in a competition setting. A frequently used parameter that often corresponds well with instantaneous energy requirement is the propulsive power (*P*_prop_) generated by the athlete. For some endurance sports, like cycling, *P*_prop_ can be measured directly, and metabolic energy requirements are approximately linearly related to *P*_prop_ (Ettema and Lorås, 2009). Hence, if *P*_prop_ can be linked to metabolic power in skiing (Millet et al., 2003; Sandbakk et al., 2011; Karlsson et al., 2018), in-field measurements of *P*_prop_ would be useful to further our understanding of the physiological demands experienced by a competitive skier. Therefore, several studies have attempted to calculate *P*_prop_ during cross-country skiing, based either on position measurements (Sandbakk et al., 2011; Swarén and Eriksson, 2017), or simulation of skiing performance (Moxnes and Hausken, 2008; Carlsson et al., 2011; Moxnes et al., 2013, 2014; Sundström et al., 2013). These studies all use the principle of power balance, as outlined by van Ingen Schenau and Cavanagh (1990). However, no studies are available where *P*_prop_ has been measured continuously throughout a cross-country ski race with a duration longer than sprint skiing. Furthermore, no previous studies have critically evaluated the accuracy achieved when applying the power balance principle to cross-country skiing. The aims of this study were (1) to develop a procedure for estimating the propulsive power generated during roller-skiing using small non-intrusive sensors (GNSS and IMUs) attached to the athlete and evaluate its limitations; and (2) to utilize this procedure to determine the propulsive power generated by high-level skiers during a simulated distance race.

### Theoretical background

As stated by van Ingen Schenau and Cavanagh (1990), the *P*_prop_ is equal to the rate of change in mechanical energy (*E*_mech_) of the system and the work done on the environment (*W*_env_). In cross-country skiing *P*_prop_ is customarily estimated by modeling the skier and his/her equipment as a point mass (Moxnes and Hausken, 2008; Carlsson et al., 2011; Moxnes et al., 2013, 2014; Sundström et al., 2013; Swarén and Eriksson, 2017). Under this assumption, the mechanical energy is the sum of translational kinetic energy and the gravitational potential energy. Work done on the environment is primarily due to ski/snow-friction forces (or rolling resistance, denoted **F**_f_) and the aerodynamic drag force (**F**_d_). This is summarized in Equation 1:

(1) $$\begin{array}{l} P_{\text{prop}}={\dot{E}}_{\text{mech}}+{\dot{\text{W}}}_{\text{env}}\\ \;=\frac{\left(m\dot{v}-{\text{F}}_{g}-{\text{F}}_{\text{d}}-{\text{F}}_{\text{f}}\right)\cdot \text{v}.}{\text{Point mass assumption}} \end{array}$$

In Equation 1 *m* refers to the total mass of the system (the sum of body mass and equipment), **v** is the velocity of the center of mass (COM), $\dot{v}$ is the COM acceleration, and **F**_g_ is the gravitational force. Furthermore, the magnitude of the propulsive force (*F*_prop_), i.e., the force in the skiing direction that is not due to gravity or frictional forces (air drag, ski/snow-friction or rolling friction) is calculated using *F*_prop_ = *P*_prop_·|*v*|^−1^ (Carlsson et al., 2011).

For skiing and roller skiing applications **F**_f_ has commonly been modeled using the Amonton-Coulomb equations (Carlsson et al., 2011; Moxnes et al., 2013, 2014; Sundström et al., 2013; Swarén and Eriksson, 2017). This friction model is attractive because of its simplicity, but it is unable to capture complex ski-snow interactions (Bowden and Hughes, 1939; Buhl et al., 2001; Theile et al., 2009), and **F**_f_ may change considerably over the course due to changing snow conditions. This challenge can be partially overcome using roller skis, which have a more constant coefficient of rolling resistance, except during the warm-up period (Ainegren et al., 2008). Another limitation is that during turns or when employing ski-skating techniques the skis (or roller skis) are actively pushed in the mediolateral direction, which causes shear forces. One study has investigated how the rolling resistance of a roller ski was affected by shear forces occurring during the ski push-off (Sandbakk et al., 2012). They concluded that the ratio of the rolling resistance force to the vector sum of shear and compression forces varied by <2% for ski angles up to 45°. This finding suggests that when evaluating rolling resistance during roller ski skating, shear forces (caused by ski push-off or centripetal forces during a turn) can be added to the normal force, at least for the wheel type assessed by Sandbakk et al. (2012).

Work done against the aerodynamic drag force has conventionally been modeled using the drag equation from fluid dynamics (Carlsson et al., 2011; Moxnes et al., 2013, 2014; Sundström et al., 2013; Swarén and Eriksson, 2017):

(2) $$F_{d}\;=-\frac{1}{2}\rho v_{f}^{2}\cdot AC_{D}(\text{Re})\cdot {\hat{v}}_{f}.$$

In Equation 2, ρ denotes the air density, ***v***_f_ denotes the velocity relative to the air, and $\hat{\text{v}}$_f_ the unit vector along ***v***_f_. The drag area (*AC*_*D*_) is the product of the frontal area *A* of the athlete and equipment and the drag coefficient (*C*_*D*_), which depends on the shape and surface material properties of the object in the air flow. *C*_*D*_ also depends upon the Reynolds number:

(3) $$\begin{array}{r} \begin{array}{c} \text{Re}=\frac{\rho Lv_{f}}{\mu }. \end{array} \end{array}$$

In Equation 3, ρ denotes the fluid's density, *L* is the characteristic length of the object, and μ is the dynamic viscosity. *C*_D_ is relatively constant when the flow is turbulent, except for a sharp drop when the boundary layer transitions from semi-turbulent to fully turbulent flow, resulting in a narrower wake behind the object (Spurk and Aksel, 2008). This phenomenon usually occurs at Re around 2 × 10^5^ and is well studied for simple blunt bodies (Achenbach, 1968; Spurk and Aksel, 2008). The drop in *C*_D_ has also been shown to exist for athletes while roller skiing (Spring et al., 1988) and during wind tunnel simulations of ice skating (van Ingen Schenau et al., 1982), where the transitions occurred at about 5 and 10 m·s^−1^, respectively. The magnitude of the change in *C*_D_ varies considerably between different studies. Data from Achenbach (1968) and van Ingen Schenau et al. (1982) showed that *C*_D_ was reduced to about 30–40% of its quasi-stable value for Re < 10^5^, while data from Spring et al. (1988) implied a decrease to about 10%. There are two more challenges when estimating **F**_d_ from Equation 2: (i) wind velocity must be known in order to find *v*_f_; and (ii) the drag area depends on the skier's posture and (indirectly) on skiing speed, through the Reynolds number. Previous studies have estimated *AC*_D_ as constant (Swarén and Eriksson, 2017), or as a step or smooth function of skiing speed using allometric scaling based on body mass (Sundström et al., 2013; Moxnes et al., 2014), and only one study has investigated the effect of non-zero wind velocity (Moxnes et al., 2014). These simplifications are often necessary because direct measurement of instantaneous wind field and drag area are challenging or impossible in the field. Nonetheless, the error arising from these approximations has rarely been addressed.

## Materials and methods

### Participants and study design

The data presented in this study were collected over three test days on the roller skiing course at Holmenkollen, Oslo, Norway. The topography of the course is similar to race courses used in competitive cross-country skiing on snow (height difference 51 m, maximum climb 32 m, total climb 166 m). Eight skiers (seven cross-country skiers; FIS point range of 13–117, and one biathlete) volunteered for the study and gave their written consent to participate. The study was approved by the ethics committee at the Norwegian School of Sport Sciences and the Norwegian Centre for Research Data, and was conducted according to the Declaration of Helsinki.

**Figure 1:**
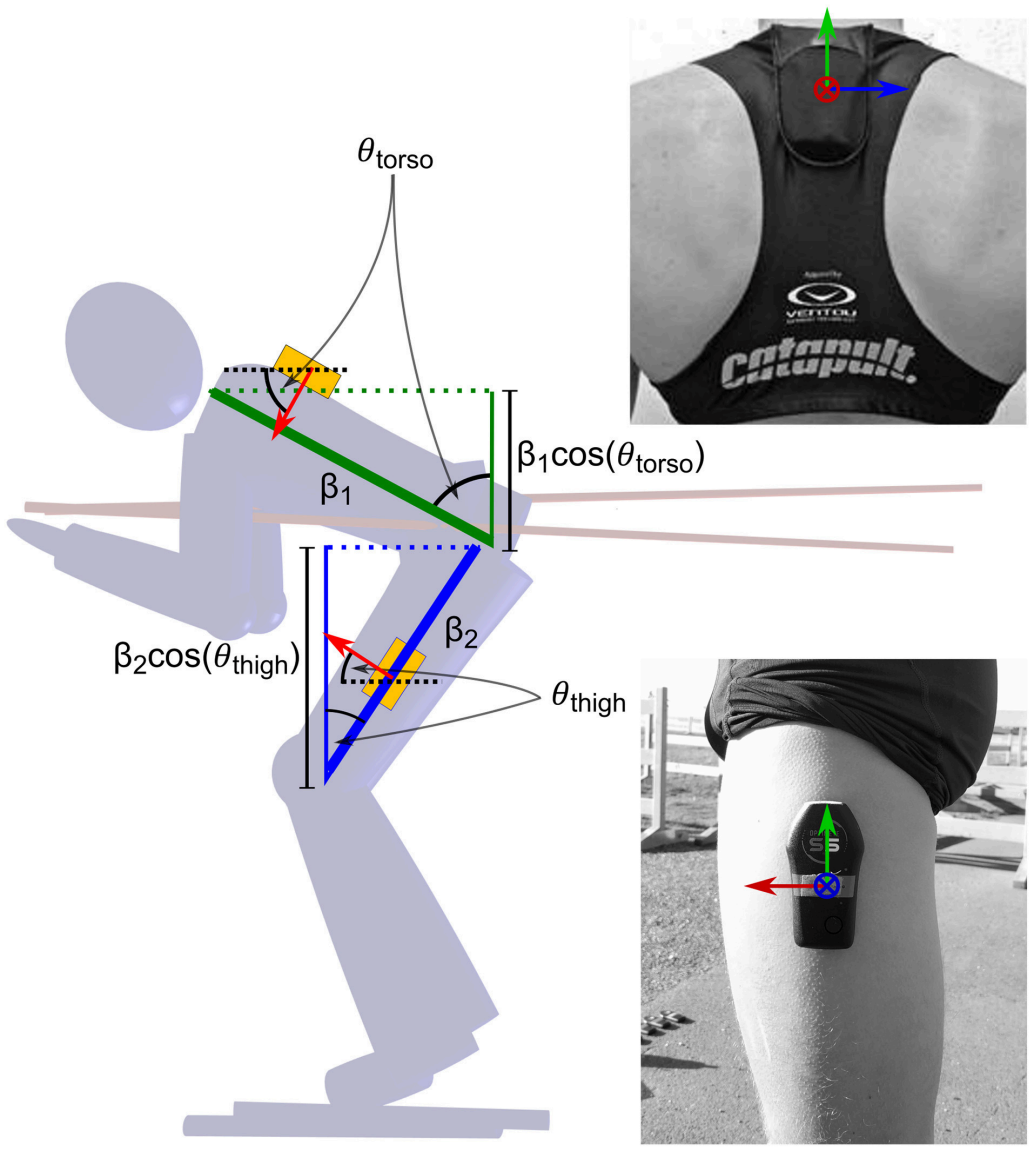
Positioning of the two IMUs on the athletes' body and motivation for the frontal area model described in Equation 5. One IMU was positioned approximately at the level of the third thoracic vertebra, the other was taped laterally on the thigh approximately 10 cm inferior to the trochanter. The axes were aligned with the x-axis (blue) in the mediolateral direction and the y-axis (red) in the anterior direction. The z-axis (green) was aligned with gravity when the athletes were in a standing posture, as described in the text. The frontal areas of the torso and thighs scale approximately with the cosine of the pitch angles θ _k_ = atan(*a*_y, k_/*a*_z, k_), where *a*_y, k_ and *a*_z, k_ are the smoothed accelerometer outputs along the y and z-axis respectively. *k* represents the thigh or torso location.

The participants were asked to complete a test race consisting of three laps of a 4.5 km course in the shortest time possible. The test race was arranged as a time trial and the participants were instructed to the use skating techniques. All participants used the same model of roller skis (Swenor Skate Long, wheel type 2), and wore tight-fitting clothing. Each participant was equipped with two identical position tracking devices (Catapult Optimeye S5, mass 67 g) consisting of a 10 Hz standalone GNSS-module and a 9-axis inertial measurement unit (accelerometer, gyroscope and magnetic field measurements). One of the receivers, carried in a tight-fitting vest, was positioned at approximately the level of the third thoracic vertebra, while the other was taped laterally on the thigh approximately 10 cm inferior to the trochanter (Figure 1). Both units were attached so that the inertial sensors' local coordinate systems (*xyz*) were approximately aligned with the *x-*axis directed the mediolaterally to the right (in the skiing direction) and the *y-*axis in the anterior direction. Prior to the test race the weight of the athletes and equipment (including ski boots, roller skis, ski poles and helmet) was recorded, and the participants performed calibration measurements for the drag area model, as described in section Frontal Area. After completing these measurements, the athletes warmed up for approximately 30 min before the race started.

To allow more accurate determination of vertical position, the GNSS positions from the receivers carried by the athletes were mapped onto a common trajectory that was determined from kinematic position tracking using a more accurate differential GNSS (receiver: Alpha-G3T, antenna: GrAnt-G3T, Javad, USA). These measurements had an expected accuracy <5 cm (Gilgien et al., 2014b) when double difference ambiguities were fixed. The ambiguities could not be solved for five short sections of the course, resulting in a substantially reduced accuracy in these sections (expected errors >1 m Gilgien et al., 2014b). These sections are clearly marked in the results.

### Environmental conditions

Air temperature and pressure were recorded during the race for each day of testing. Wind velocities (measured 10 m above the ground) were retrieved hourly from weather stations located approximately 2.3 km (Tryvann) and 3.7 km (Blindern) from the roller skiing course (www.met.eklima.no, Meteorological Institute of Norway, Oslo, Norway). The wind velocities were averaged and corrected to 1 m height above the ground using the log wind profile relationship (Oke, 1978) with a roughness length of 0.1 m, resulting in wind speeds exactly half of the data measured 10 m above the ground.

### Data processing and filtering

The GNSS positions and inertial sensor data from the receivers mounted on the athletes were exported using Catapult Sprint software version 5.1.7 at sampling frequencies of 10 and 100 Hz, respectively. Differential GNSS positions for the reference trajectory were calculated using the kinematic algorithm of the geodetic post-processing software Justin (Javad, San Jose, CA, USA). All other analyses were performed using Matlab R2017a (MathWorks, Natick, MA, USA).

The reference trajectory was resampled to equidistantly (1 meter) spaced points, and then filtered using smoothing splines weighted by their fixed/float status (Skaloud and Limpach, 2003) using a smoothing parameter of *p* = 0.02. The GNSS positions obtained from the receivers mounted on the athletes were filtered with a second order bidirectional Butterworth low pass filter with a cutoff frequency of 0.3 Hz, which removed the high frequency components that could not be attributed to the center of mass trajectory due to the receiver's antenna location. The cutoff-frequency was chosen based on a frequency-analysis of motion capture data from skiing using the V1 and V2 techniques. Only the GNSS positions from the receiver on the torso were used for skier position calculation. Vertical position (*z*) was obtained by mapping the horizontal plane position from the standalone GNSS receiver carried by the athlete onto the 3D reference trajectory. This was achieved by minimizing horizontal plane Euclidean distance from the position measurement of the standalone GNSS receiver to any point on the reference trajectory. After filtering and projection of the standalone GNSS positions onto the reference trajectory the trajectories were down-sampled to a frequency of 1 Hz prior to work rate calculations, to limit the computational load. Finally, external work rate was filtered using a 5 second bidirectional moving average filter.

### Mechanical energy

For calculation of mechanical energy, a point mass *m* equal to the combined mass of the athlete and his equipment was utilized. With this approach the gravitational potential energy is *mgz*, where *g* = 9.81m·s^−2^ is the acceleration due to gravity and *z* the vertical position. The kinematic energy is 0.5m|**v|**^2^, where **v** is the skiing velocity. The skiing velocity was determined by differentiating the horizontal plane positions from the standalone GNSS receiver carried by the athlete and the vertical component of velocity was calculated using the using the mapped vertical position. Velocity was calculated using a five-point finite difference algorithm (Gilat and Subramaniam, 2008).

### Rolling resistance

Rolling resistance was measured individually for each athlete using a towing test on the roller skiing treadmill at the Norwegian School of Sport Sciences, as described by Hoffman et al. (1990). The coefficient of rolling resistance (*C*_*rr*_) was 0.0225 ± 0.0009 (mean ± standard deviation). The rolling resistance of one of the pairs of roller skis on asphalt (determined by a towing test) was the same as on the treadmill surface, in agreement with the findings of Myklebust (2016), and the former were therefore used in the calculations of work against rolling resistance. The roller skis were assumed to move the same distance as the GNSS antenna, and centripetal forces (**F**_c_, caused by the course's curvature) were added to the normal force opposing gravity (**N**_g_), following the findings of Sandbakk et al. (2012). Hence, the work rate against rolling resistance was estimated using the following equation:

(4) $$\begin{array}{r} \begin{array}{c} {\dot{W}}_{f}=C_{rr}\cdot |{\text{N}}_{g}+{\text{F}}_{c}|\cdot v. \end{array} \end{array}$$

In Equation 4, **N**_g_ was defined as minus the component gravity perpendicular to the course's normal vector, and the course was assumed to be level in the mediolateral direction. Centripetal force was calculated using **F**_c_ = *m|****v****|*^2^·**K**, where **K** = −**v**×**(v**×**$\dot{\text{v}}$)/***v*^4^ is the track curvature vector (Dooner, 2012).

### Air drag model

Air drag was determined from Equation 2, with the drag area as defined in the next two paragraphs. Air density ρ_air_ = *p*·$R_{\text{specific}}^{-1}$*T*^−1^ was calculated from measured air pressure using the ideal gas law with *R*_specific_ = 287.058 J·kg^−1^·K^−1^, and *p* and *T* equal to the measured air pressure and temperature, respectively. Dynamic viscosity (μ_air_) was calculated as a function of air temperature using Sutherland's formula, as described by Canuto et al. (2007). Wind velocity was defined as described in section Statistics and was subtracted from the skiing velocity vector. Finally, the power dissipated through air drag was the dot product **F**_d_·**v**.

#### Frontal area

**Figure 2:**
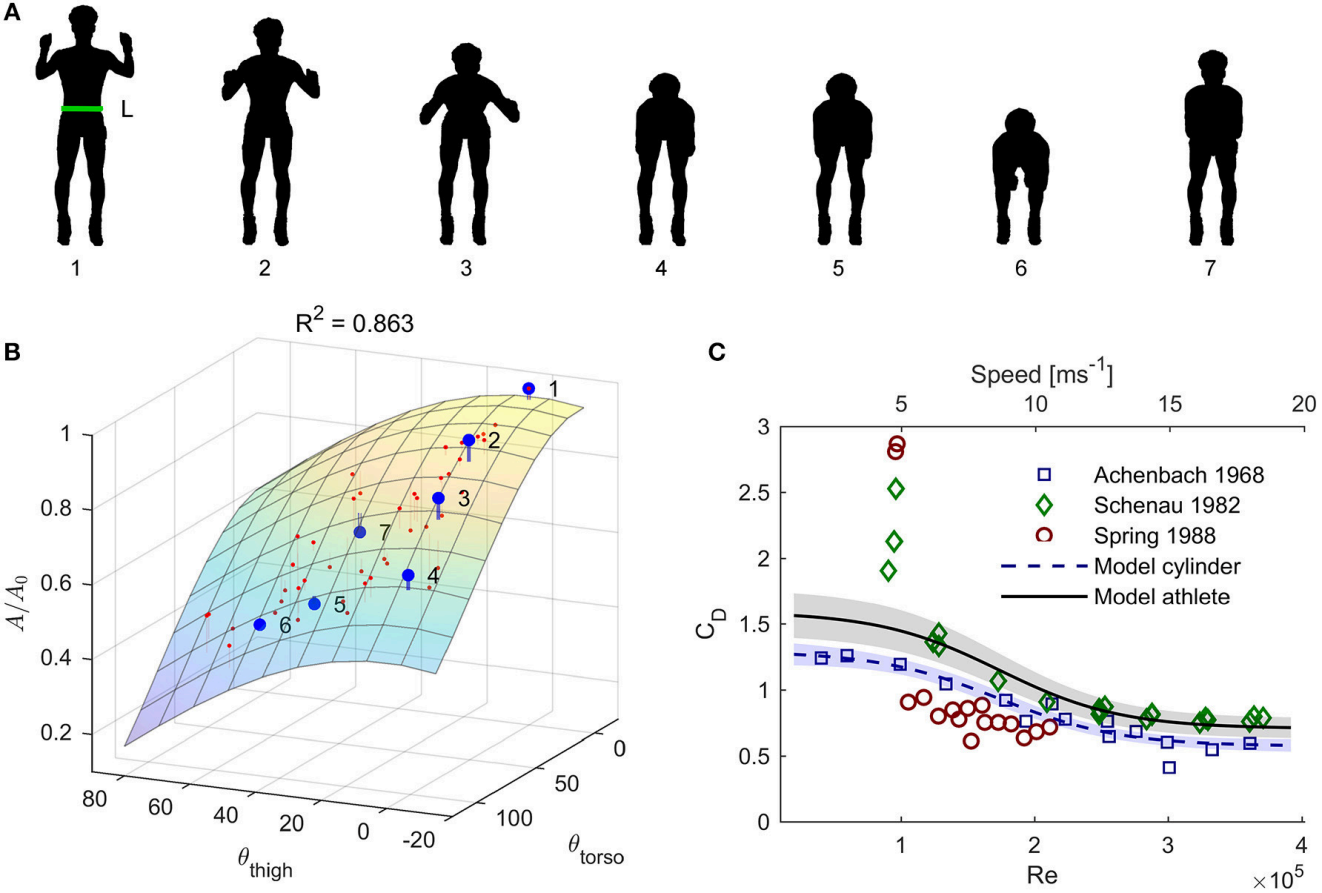
**(A)** Postures used to calculate frontal area. The first pose for each athlete was used to calculate the characteristic length *L* (Equation 3), which was defined as the width at the height of the pose's center of mass (green line). **(B)** Model for the frontal area *A* based on the postures in **(A)** and Equation 5. The data points are enumerated to match the postures in **(A)**, and show the measurements from one subject. Red dots indicate measurements from all other subjects. **(C)** Model (Equations 7, 8) for the drag coefficient of a skier as a function of the Reynolds number (solid black line). Blue squares: data from Achenbach (1968); Green diamonds: data from van Ingen Schenau et al. (1982); Red circles: data from Spring et al. (1988); Dashed blue line: model fitted to Achenbach's measurements. The upper and lower x-axis were related by Equation 3 using atmospheric conditions as specified in van Ingen Schenau et al. (1982) and *L* = 0.3 m. Shaded areas indicate the models' standard deviations, and were obtained using Monte Carlo simulations (*N* = 5,000) sampled from the distributions specified in the statistics section.

The skiers' frontal area *A* was approximated continuously during the entire trial using the accelerometer data provided by the sensors mounted on the torso and thigh. First, the accelerometers' coordinate frames were rotated so that the z-axis was parallel to the gravity vector during a static standing pose (Figure 2A, pose 1), and the x-axis was directed mediolaterally. This was achieved by performing two successive rotations, the first canceling lateral tilt and the second canceling forward tilt (Myklebust et al., 2015). Second, the signals were filtered with a 2 second bidirectional moving average filter. Third, the smoothed pitch angles θ _thigh_ and θ _torso_ were calculated using tan(θ) = *a*_*y*_/*a*_*z*_, where *a*_*y*_ and *a*_*z*_ denote the smoothed accelerometer outputs (in a standing posture) along the forward and vertical directions. With this definition, the pitch angle represents a rotation about the IMU's *x*-axis that will align the *y*-axis with the horizontal plane. As shown in Figure 1, the frontal areas of the torso or thigh segments will scale approximately with the cosine of this angle. Therefore, θ _thigh_ and θ _torso_ were used to predict the frontal area *A* of the athlete using the following equation:

(5) $$\begin{array}{r} \begin{array}{c} \frac{A-A_{\text{equip}}}{A_{0,j}}=\beta_{0}+\;\beta_{1}\cos\left(\theta_{torso}\right)+\beta_{2}\cos\left(\theta_{thigh}\right). \end{array} \end{array}$$

Here *A*_0, *j*_ denotes the frontal area of athlete *j* while standing upright, as shown in Figure 2A, and *A*_equip_ = 0.045 m^2^ was a constant that was added to represent the average frontal areas of the roller skis and ski poles. The three parameters β_0, 1, 2_ were determined using multiple linear regression with cos(θ _thigh, (i, j)_) and cos(θ_torso, (i, j)_) as predictors, and 56 frontal areas *A*_(i, j)_ (i.e., seven frontal areas per participant) with different postures as the dependent variable (Figures 2A,C). The 56 frontal areas were calculated from digital images (resolution 3,264 × 4,928 pixels) taken of the skiers prior to the trial, and the pixel size was determined using an object of known length placed directly lateral to the athlete. The characteristic length *L*_*j*_ in Equation 3 was defined as the width of athlete *j* in the pelvis/abdomen region and was determined from the first of the 7 images (Figure 2A).

#### Determination of the frontal area by allometric scaling

We also tested a drag area model that simplifies data collection and analysis, because it does not require measurements of frontal areas or measurements from inertial sensors. First, the estimated frontal areas determined from Equation 5 were normalized by *m*^2/3^, where *m* was body mass and 2/3 is the allometric scaling exponent (Günther, 1975; Bergh, 1987). Second, the normalized frontal areas were assumed to be a sigmoid function of skiing speed *v*, as previously assumed by Moxnes et al. (2014) and Sundström et al. (2013). To model this sigmoid shape, we defined a logistic function:

(6) $$\begin{array}{r} \begin{array}{c} \frac{A\left(v\right)}{m^{2/3}}=\gamma_{1}-\frac{\gamma_{2}}{1+e^{-\left(\frac{v}{\gamma_{4}}-\gamma_{3}\right)}}. \end{array} \end{array}$$

The four-parameter vector γ was determined by minimizing the sum of the squared residuals from the normalized estimates of drag area (A·*m*^−^^2/3^) determined from Equation 5 using the Levenberg-Marquardt algorithm. Hence, once γ was established, the only necessary input was body mass and skiing speed.

#### Drag coefficient

The drag coefficient *C*_D_ was modeled as a function of the Reynolds number (Re) using a logistic function fitted to the data from Achenbach (1968). Specifically, the four-parameter vector α was determined by minimizing the sum of the squared residuals between measurements and the model in Equation 7 (below) using the Levenberg-Marquardt algorithm (implemented in Matlab's Statistics and Machine Learning toolbox):

(7) $$\begin{array}{r} \begin{array}{c} {C_{D}}^{\prime }\left(\text{Re}\right)=\alpha_{1}-\frac{\alpha_{2}}{1+e^{-\left(\frac{\text{Re}}{\alpha_{4}}-\alpha_{3}\right)}}. \end{array} \end{array}$$

Only measurements with Reynolds numbers in the range [4 × 10^4^, 5 × 10^5^], which corresponds to conditions relevant to cross-country skiing (speeds in the range 2–25 m·s^−1^, assuming μ·ρ^−1^ = 1.5 × 10^−5^m^2^·s^−1^ and L = 0.30 m) were used for the fitting procedure (Figure 2C). In the second step, the model in Equation 7 was scaled to match the mean drag coefficients of speed skaters measured at 12 m·s^−1^ by van Ingen Schenau et al. (1982). This was done by calculating the Reynolds number (Re_Schenau_) for the conditions described in van Ingen Schenau et al. (1982), and then applying the following equation:

(8) $$\begin{array}{r} \begin{array}{c} C_{D}\left(\text{Re}\right)=\;\frac{C_{D,\text{Schenau}}\;}{{C_{D}}^{\prime }\left({\text{Re}}_{\text{Schenau}}\right)}C_{D}^{{}^{\prime }}\left(\text{Re}\right). \end{array} \end{array}$$

### Tuck position

**Figure 3:**
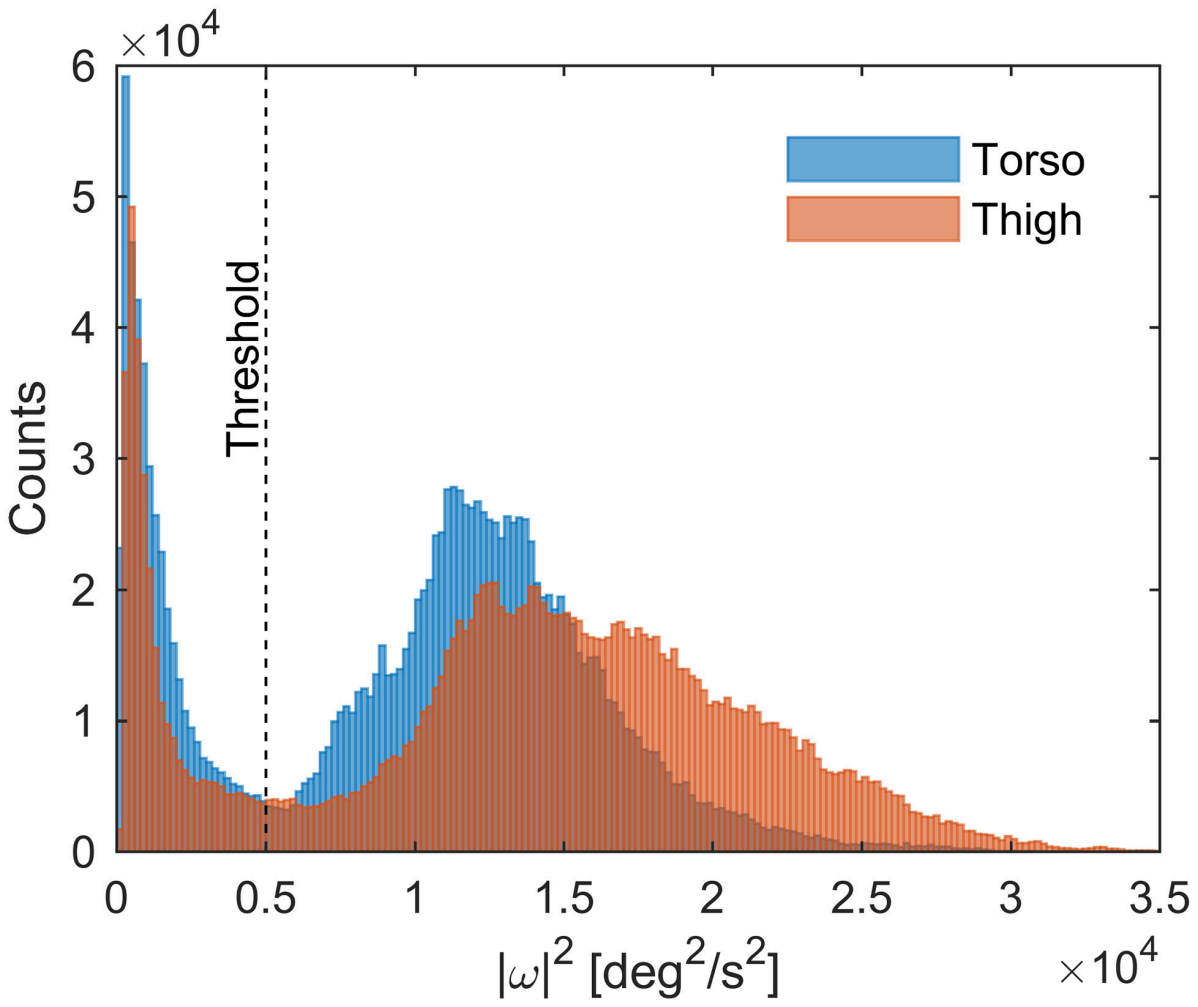
Histogram of the squared magnitude of gyroscope measurements (angular velocity) during skiing for all athletes. The distinct distribution minimum at about 5,000^°2^s^−2^ indicates the transition from the tucked position, where movements of both torso and thigh are small, to skiing techniques that generate propulsion. In the current study, the athletes were assumed to be in the tucked position when |ω|^2^ of both torso and thigh were <5,000^°2^·s^−2^.

When in the tucked position, skiers do not generate any significant propulsive force. As all cross-country skiing techniques (except the tucked position) require substantial rotation of both the thorax and thigh segments, measurements of the segments' angular rates were used to determine when the athletes were in the tucked position. Specifically, the squared magnitude of the angular velocity vector from the gyroscopes was used as a decision criterion. These signals were filtered with a 2 second bidirectional moving average filter, and athletes were defined to be in the tuck position when both devices (on thigh and thorax) showed values smaller than 5,000^°2^·s^−2.^ This threshold was determined by inspecting the signal distributions (Figure 3). *P*_prop_ was set to zero when the athletes were in the tucked position.

### Statistics

Confidence intervals for the calculated work rates were determined using a Monte Carlo approach. The effect of changing wind velocity and errors in drag area and rolling resistance were modeled by sampling from distributions as described in this section. 2,500 Monte Carlo samples were used for each of the participants.

Wind speed was assumed to be Rayleigh distributed (Dorvlo, 2002) with an expectation value equal to the hourly average wind speed after correction for height above ground, and averaged over the two measurement stations. Wind direction was assumed to be normally distributed with an expectation value equal to the hourly average and a standard deviation of 25 degrees.

Errors in frontal area estimates (Equation 5) were assumed to be ~***N***(0, $\sigma_{\text{resid}}^{2}$), σ_resid_ being the standard deviation of the model residuals. The distribution of the coefficients α in Equation 7 was assumed to be ~***N***(α_opt_, $\sigma_{\text{opt}}^{2}$), where α_opt_ and σ_opt_ were the coefficient estimates and covariance matrix obtained from the optimization procedure. *C*_D, Schenau_ was defined to be ~***N****(*0.872, 0.079^2^*)*, which corresponds to the mean and standard deviation of the findings in Table 1 of van Ingen Schenau et al. (1982).

The coefficient of rolling resistance was assumed to be normally distributed with an expectation value equal to the measured values from the treadmill towing test and standard deviation 2.3 × 10^−3^, which was the standard error of the measurements from the towing test on asphalt. Asphalt values were used for the standard deviation, since these were assumed to better represent the variability of field conditions.

The method's accuracy was defined as the pooled standard deviation of *P*_prop_ and *F*_prop_ obtained from the Monte Carlo simulation. The method's sensitivity was assessed by comparing inter-athlete and intra-athlete differences in *F*_prop_ to the method's accuracy. This was achieved by calculating the empirical cumulative distribution function of the inter-athlete or intra-athlete standard deviation (i.e., the lap-to-lap variability) of *F*_prop_. The method was considered suitable to discriminate differences in *F*_prop_ if the standard deviation was greater than the measurement accuracy for >90% of the measurements. To test the method's sensitivity in optimal conditions, the Monte Carlo simulation was run again with zero wind speed. All other parameters were kept constant.

## Results

### Physiological aspects

**Figure 4:**
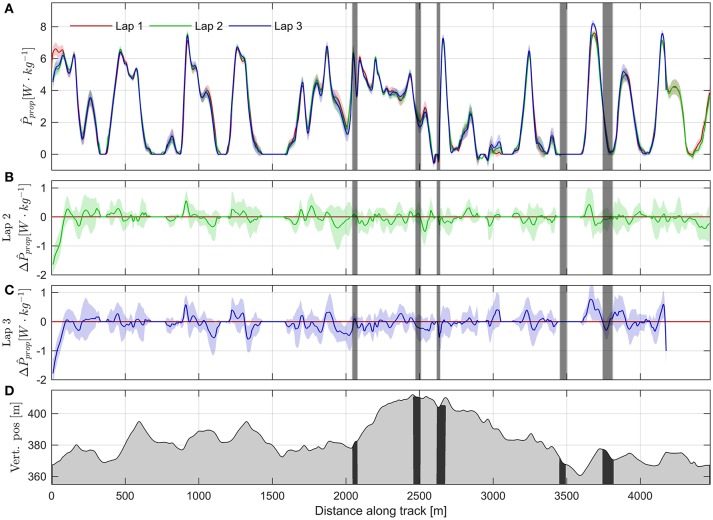
**(A)** Propulsive power normalized to body mass plotted over distance along the course for the three laps (lap 1 red, lap 2 green, lap 3 blue). Colored regions around the solid lines show the standard deviations from the Monte Carlo simulation. Vertical gray shading indicates regions where double differenced ambiguities were float. The Monte Carlo simulation does not account for the reduced accuracy in these regions. Negative values of *P*_prop_ occurs when *F*_prop_ < 0 and could be caused by either active breaking of the athlete, or by measurement error. **(B)** Mean difference in propulsive power between lap 2 and lap 1. Colored shaded region shows the 95% CI. **(C)** Same as B, but for the difference between lap 3 and lap 1. **(D)** Altitude profile of the competition course. Black regions correspond to the regions where double differenced ambiguities were float.

A graphical presentation of *P*_prop_ as a function of distance traveled is provided in Figure 4, which clearly shows that there were substantial variations in *P*_prop_ throughout the test race. *P*_prop_ was 3.35 ± 0.23 W·kg^−1^ when averaged over the race duration, and 4.18 ± 0.41 W·kg^−1^ when omitting measurements where the athletes were in the tucked position (termed active propulsive power, Swarén and Eriksson (2017)). Peak *P*_prop_ was typically attained on the last major ascent during the final lap (at 3,600–3,730 m from the start of the lap in Figure 4). The average of the athletes' peak *P*_prop_ was 8.35 ± 0.63 W·kg^−1^. When comparing *P*_prop_ at the same positions along the course, lap-to-lap differences were small, except for a distinct starting spurt for the initial 200 meters of the first lap (Figures 4B,C). *P*_prop_ was also higher in the last two uphills of the third lap (3,600–3,730 and 4,120–4,170 m) compared to the first lap, indicating an end spurt (Figure 4C).

**Figure 5:**
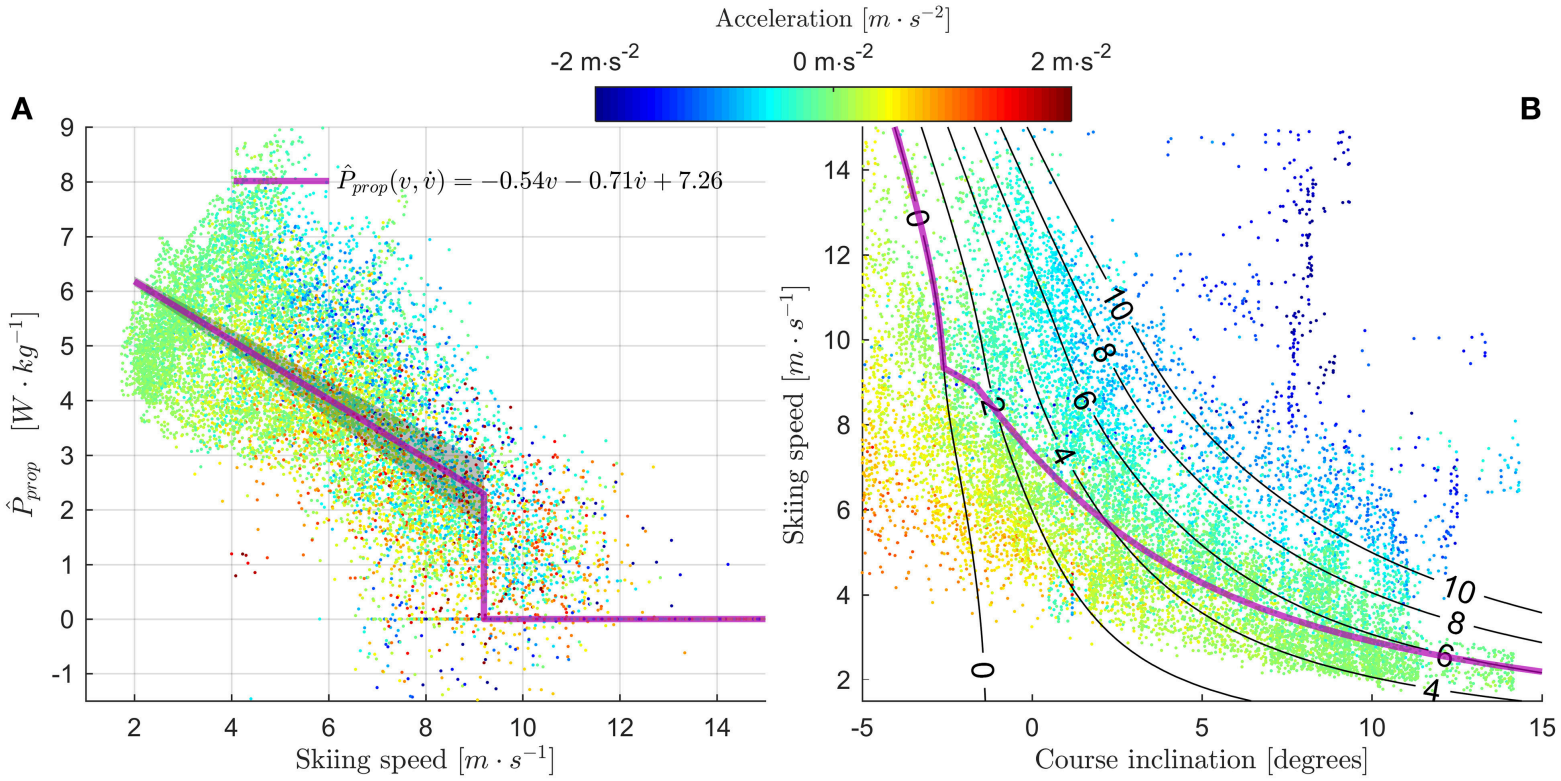
**(A)** Propulsive power (normalized to body mass) was approximately linearly related to skiing speed (*v*) and acceleration ($\dot{v}$) in the skiing direction. Data from all athletes are included in the figure, and data points are color coded by $\dot{v}$, as indicated by the colorbar above the figure. The magenta-colored line is the least squares regression fit to samples where the athlete was not in the tucked position. The shaded region indicates the measurement error (SD), see Figure 7 for details. **(B)** Plot of skiing speed vs. the course inclination. Data points are color coded as in panel **(A)**. The black lines indicate constant propulsive power (in W·kg^−1^), assuming constant skiing speed ($\dot{v}$ = 0). The magenta line shows the steady state skiing speed obtained from the regression line in (A). It was defined as the real root of the 3rd degree polynomial obtained by replacing *P*_prop_ with *P*_prop_(*v*,$\dot{v}$) in Equation 1, and assuming a constant drag area of 0.55 m^2^ and average body mass 77.1 kg. For *v* > 9 m·s^−1^ the line was defined to follow the zero-*P*_prop_ iso-line. The figure clearly shows that data points where $\dot{v}$ ≈ 0 (green color) are distributed close to the steady-state speed line, as expected. Data points below the steady-state line have $\dot{v}$ > 0 (red), and data points above the line have $\dot{v}$ < 0. This is mainly attributed to the athletes' inertias.

**Figure 6:**
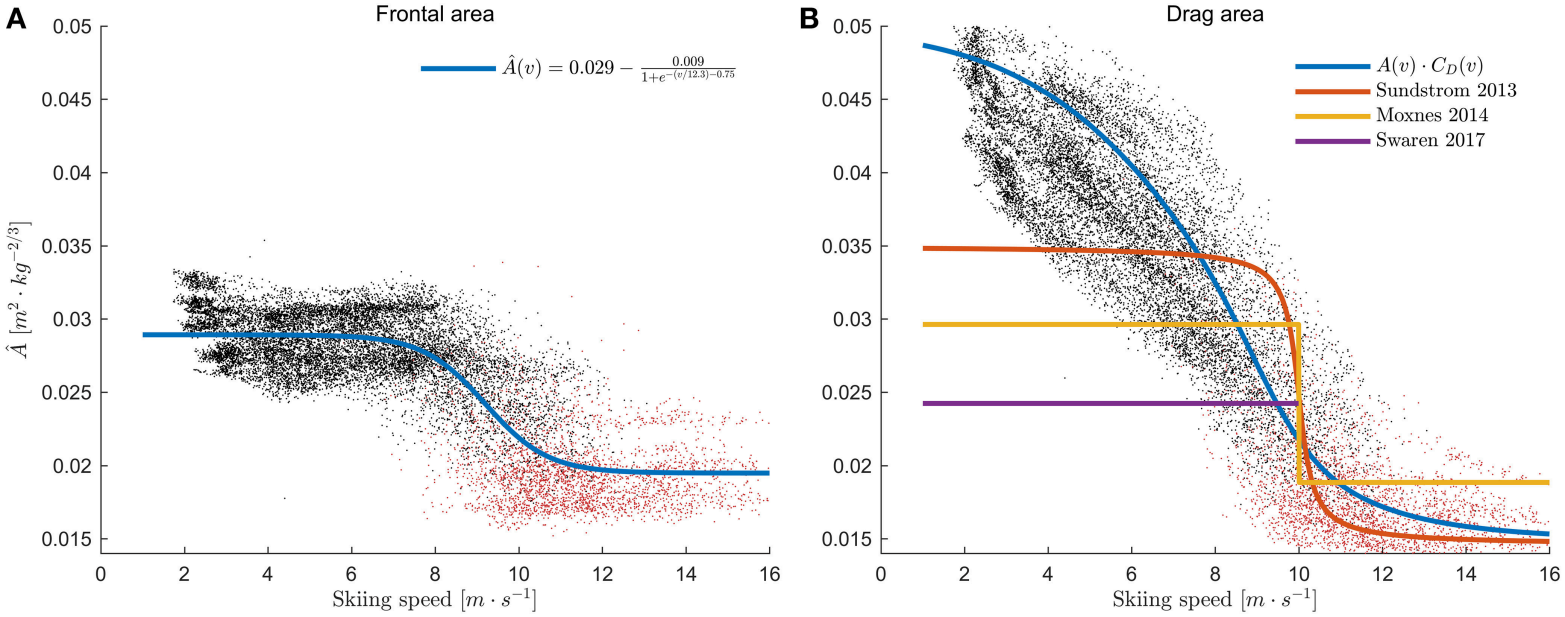
**(A)** Frontal areas (Â) estimated from the accelerometer outputs plotted over skiing speed, normalized by body mass (using an allometric scaling coefficient of 2/3). Red dots represent measurements where the skiers were in the tucked position. Blue line: logistic function fitted to the data (Equation 6). Athletes typically assumed the tucked position at skiing speeds >9.1 m·s^−1^. **(B)** Drag areas normalized to body mass (Â·*C*_*D*_) found in the current study plotted over skiing speed for all athletes. The blue line shows the product of the frontal area model from Equation 6 (plotted in panel A) and the drag coefficient (Equation 8, Figure 2). Models used by Sundström et al. (2013), Moxnes et al. (2014), and Swarén and Eriksson (2017) are included for comparison.

Overall, *P*_prop_ showed an approximately linear relationship with skiing speed (Figure 5A), except for speeds where the skiers were in the tucked position. The transition into the tucked position occurred at skiing speeds ~9 m·s^−1^ (Figure 6). Furthermore, *P*_prop_ was dependent on the acceleration along the skiing direction; a positive acceleration correlated with smaller *P*_prop_, and negative accelerations with higher *P*_prop_. This is consistent with observations from Figure 4A, where *P*_prop_ appeared to decline slightly from its initial values in the longer ascents (450–600, 1,200–1,330, 2,030–2,450 m from the start, Figure 4D). This implies that *P*_prop_ was higher when the skier decelerated at the start of a climb, and lower when accelerating over the top of a climb. A multiple least squares regression fit on *P*_prop_ using skiing speed and acceleration as predictors had the coefficients −0.54 N·kg^−1^, −0.71 N·s·kg^−1^ and intercept 7.26 W·kg^−1^ (Figure 5A), and a coefficient of determination *R*^2^ = 0.403. This indicates that a substantial fraction of the variability in *P*_prop_ could not be explained by skiing speed and acceleration alone. In Figure 5B skiing speed is plotted against the course inclination. The line predicting steady state skiing speed based on the least squares fit *P*_prop_(*v*,$\dot{v}$) was also added to the figure (see figure legend for details). The data points where the skier was close to a steady state skiing speed (i.e., |$\dot{v}$| is small) fell along the line suggested by the least squares fit. Data points far from the line suggested by the least squares fit was typically from periods with large positive acceleration (points below the line, colored red in Figure 5B) or negative acceleration (points above the line, colored blue in Figure 5B).

### Methodological aspects

In Figure 6, Frontal areas (normalized to body mass) are plotted vs. skiing speed. The frontal area changed from the typical standing posture for *v* < 8 m·s^−1^ to a tucked-position for *v* > 10 m·s^−1^ (Figure 6A). Due to the dependency of C_D_ on the Reynolds number, the behavior was more complex for the drag area, which continuously decreased almost throughout the range of skiing speeds observed during the race (Figure 6B).

**Figure 7:**
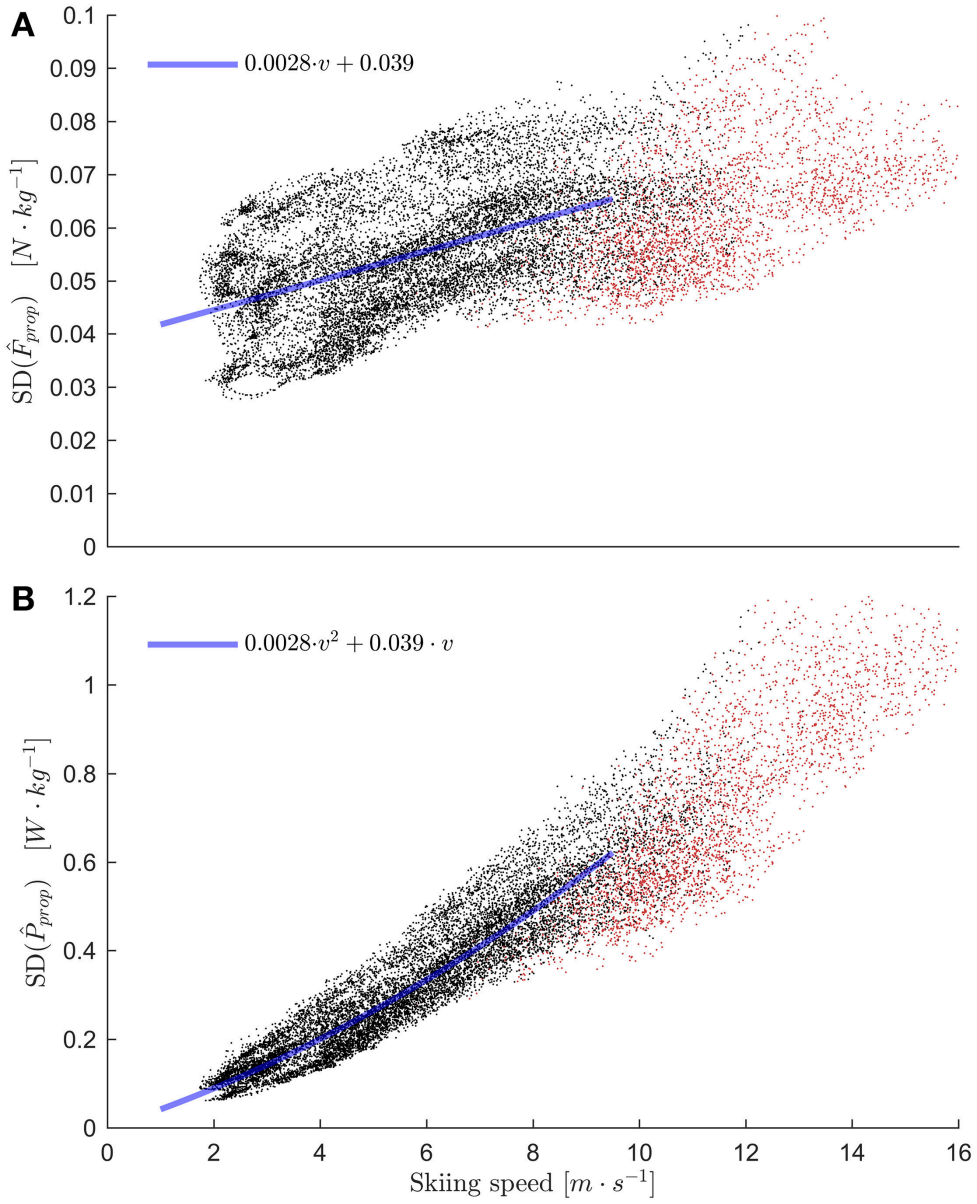
**(A)** Standard deviation of propulsive force (normalized to body mass) calculated from the Monte Carlo simulation plotted over skiing speed. The red dots are measurements when the athletes were in the tucked position. In a later step propulsive power (and force) was defined to be zero for these samples, but they are included in this figure to illustrative purposes. The blue line is the least squares linear regression line fitted to the data points where the athletes were not in the tucked position. **(B)** same as **(A)**, but for *P*_prop_. Since *P*_prop_ = *F*_prop_·*v*, the regression line from **(A)** multiplied with *v* fits well to the data.

The Monte Carlo simulation showed that errors in *F*_prop_ were on average 5.1 · 10^−2^ N·kg^−1^ and increased slowly with skiing speed (Figure 7A). The least squares regression line of *F*_prop_ with respect to skiing speed had a slope of 2.8 ·10^−3^ N·s·m^−1^·kg^−1^ and intercept 3.9 · 10^−2^ N·kg^−1^. Therefore, the error in *P*_prop_ increased curvilinearly with skiing speed, approximately following the expression 2.8·10^−3^·*v*^2^ + 3.9· 10^−2^·*v* (Figure 7B). Hence, estimates of *P*_prop_ were most accurate at low skiing speeds (~0.09 W·kg^−1^ at 2.0 m·s^−1^, close to the lowest measured speeds) and less accurate at high speeds (~0.58 W·kg^−1^ at 9.0 m·${\text{s}}_{,}^{-1}$ close to the speed when most athletes transitioned to the tucked position).

**Figure 8:**
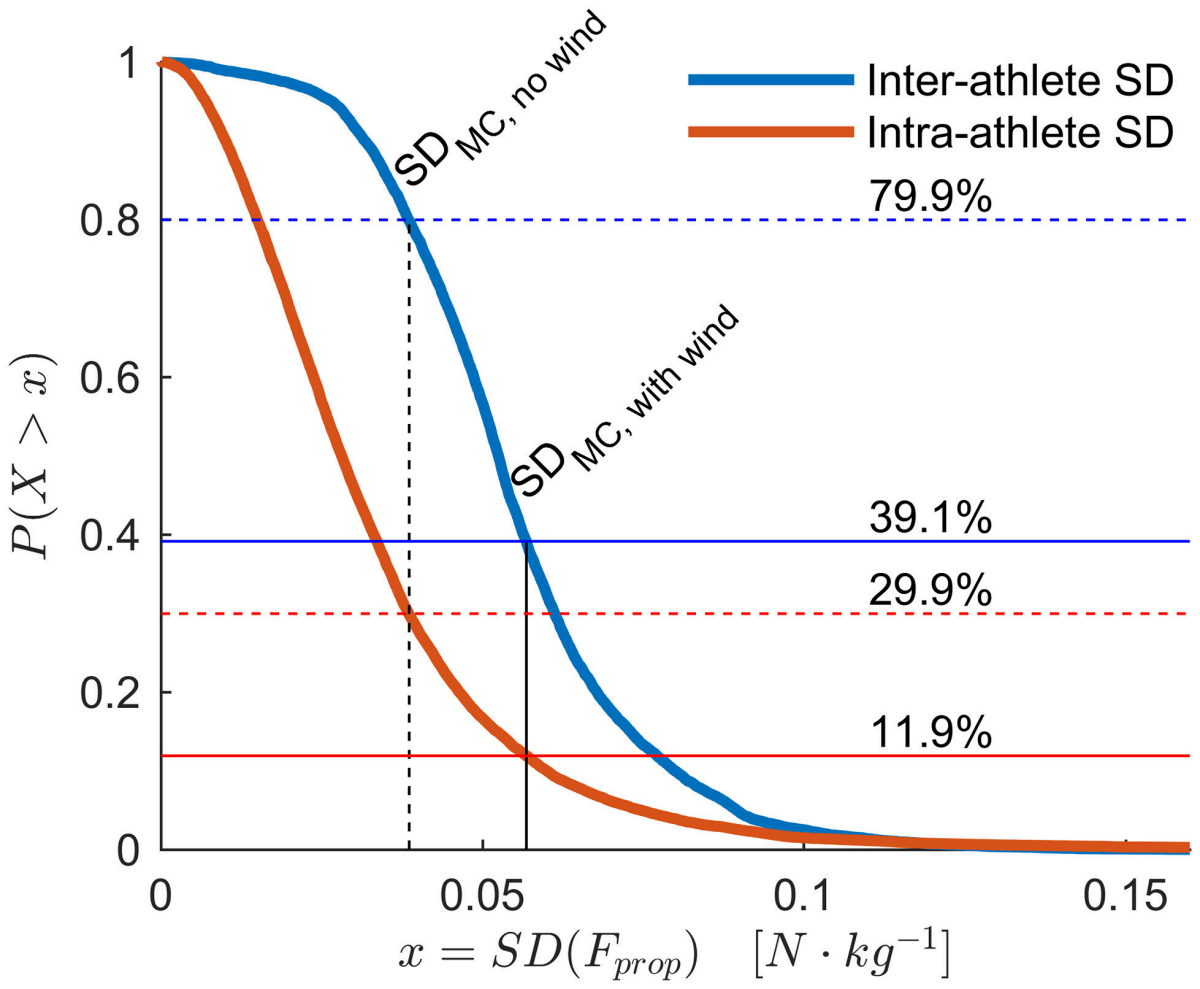
In order to discriminate the *F*_prop_ or *P*_prop_ generated by high-level athletes, the methods accuracy must be better than typical athlete-to-athlete or within-athlete differences. To that end, this figure displays the empirical complementary cumulative distribution function (ECCDF) of typical athlete-to-athlete differences in *F*_prop_ measured at the same position along the course (blue line). More specifically, it shows the between-athlete standard deviation of *F*_prop_ evaluated at every integer meter along the course, excluding measurements where the athletes were in the tucked position. The red line is the ECCDF of the within-athlete lap-to-lap standard deviation. The solid vertical line at 0.0568 N·kg^−1^ shows the mean standard deviation of *F*_prop_ from the Monte Carlo simulation, which reflects the methods typical accuracy under the specified conditions. For comparison, the dotted vertical line at 0.0385 N·kg^−1^ shows the mean standard deviation of *F*_prop_ from another Monte Carlo simulation assuming zero-wind conditions. With the measured wind conditions, inter-athlete SD was greater than the typical measurement accuracy only for 39.1% of the course, and intra-athlete SD only for 11.9% of the course. Under the zero-wind assumption, the inter- and intra-athlete differences were greater than the measurement accuracy for 79.9 and 29.9% of the course, respectively.

The method's applicability to discriminate between instantaneous inter-athlete and intra-athlete differences in *F*_prop_ is shown in Figure 8. The results show that with the test conditions in the current study, neither inter-athlete or intra-athlete differences could be reliably detected. This conclusion holds true even in zero-wind conditions, where 29.9% (intra-athlete) and 79.9% (inter-athlete) of the measurements contained differences that exceeded the measurement accuracy.

**Figure 9:**
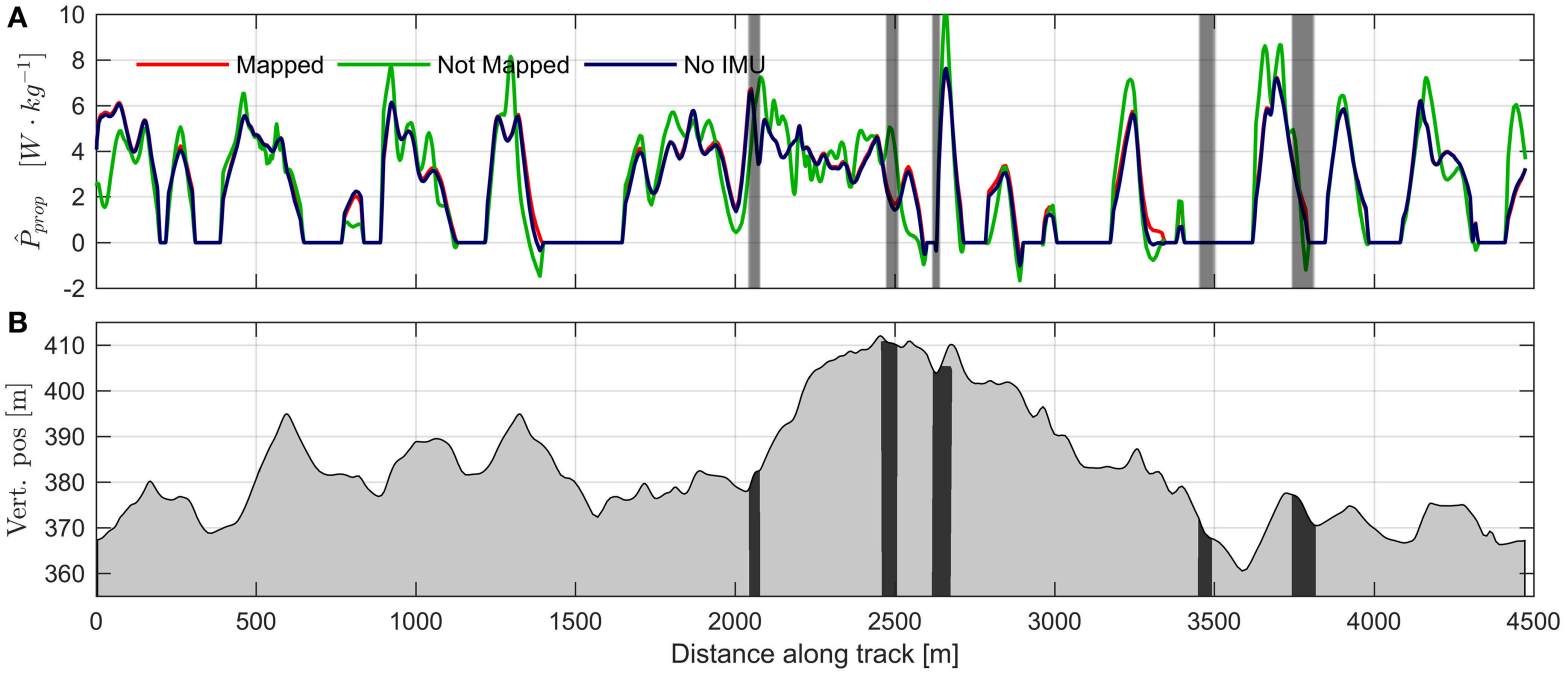
**(A)** Comparison of propulsive power calculations using mapping on dGNSS reference (blue line), calculations using only measurements from the standalone GNSS receiver (green line), and using the simplified drag area model based on body mass and skiing speed (Equation 6, red line). Data are from one lap for a single athlete. Vertical gray shading indicates regions where double differenced ambiguities were float. **(B)** Altitude profile of the competition course. Black regions correspond to the regions where double difference ambiguities were float.

For all results reported above, *P*_prop_ was calculated using vertical position measurements obtained by mapping the positions onto a reference trajectory of dGNSS measurements and using frontal area estimates from two accelerometers positioned at the thorax and thigh. Omitting the mapping procedure (by using the vertical position measurements of the standalone receivers carried by the athletes) yielded a root mean square (RMS) deviation of 1.25 W·kg^−1^ from the more accurate method of using vertical position mapped onto the dGNSS measurements. This is about 30% of mean active propulsive power. Example data for these calculations from one of the athletes are shown in Figure 9. Omitting the accelerometer measurements by using frontal areas obtained from allometric scaling (Equation 6, Figure 6) yielded an RMS deviation of 0.18 W·kg^−1^, or about 4% of mean active propulsive power (Figure 9).

Regression and curve fitting parameters for the frontal area model (Equation 5), drag coefficient model (Equation 8) and allometric scaling of frontal areas (Equation 6) are presented in Table 1.

**Table 1:** Results for the regression parameters (β) for frontal area calculation (Equation 5), parameters (α) for the drag coefficient model (Equation 7), and parameters (γ) for prediction of frontal areas using body mass and skiing speed (Equation 6).

	α		γ		β	
**Index**	**Coeff**	**95% CI**	**Coeff**	**95% CI**	**Coeff**	**95% CI**
0	1.29	[1.07, 1.52]	0.0289	[0.0289, 0.0290]	0.27	[0.21, 0.33]
1	0.72	[0.45, 0.99]	0.0094	[0.0093, 0.0096]	0.32	[0.29, 0.35]
2	4.55·10^4^	[0.93, 8.17] ·10^4^	12.2	[11.6, 12.7]	0.38	[0.31, 0.45]
3	3.82	[0.09, 7.54]	0.76	[0.72, 0.79]	–	–

### Environmental conditions

Air temperature and air pressure on the three test days ranged from 12 to 13°C and 95.1–98.1 kPa, respectively. Average hourly wind speed from the two measurements stations ranged from 2.70 to 4.48 m·s^−1^ (measured at 10 meters above ground), and average wind direction ranged from 14 to 46°.

## Discussion

**Figure 10:**
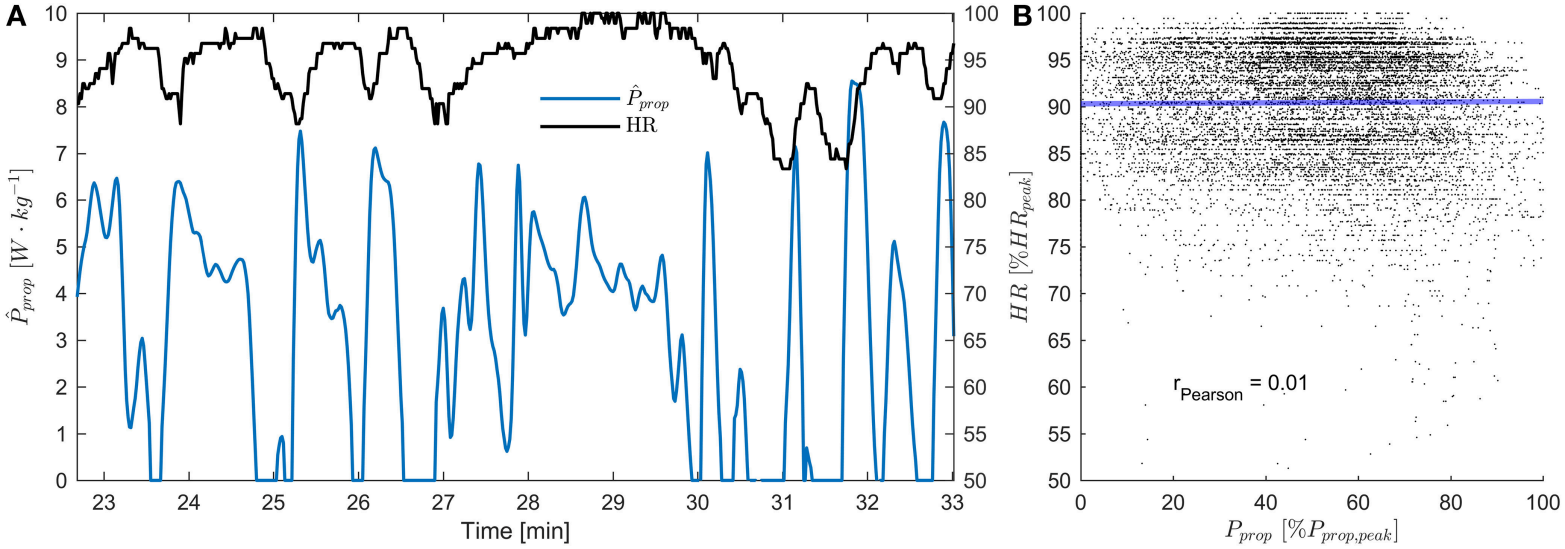
**(A)** Heart rate (HR) and *P*_prop_ measurements during the third lap for an example athlete. HR is expressed relative to the peak HR measured during the test race. **(B)** Scatter plot showing all measurements of *P*_prop_ (normalized to each athlete's peak *P*_prop_) vs. all measurements of HR. There was no significant correlation between the two parameters (r_Pearson_ = 0.01, *p* = 0.18). This indicates that changes in *P*_prop_ during cross-country ski races are too fast for heart rate to provide a valid measure of instantaneous metabolic power.

Accurate measurements of the propulsive power throughout a cross-country ski race could improve our understanding of the work requirements of cross-country skiing and other endurance sports that exhibit non-steady state power behavior (Figure 10). Calculations of propulsive power during cross-country skiing using the principle of power balance have been attempted by several authors (Moxnes and Hausken, 2008; Carlsson et al., 2011; Sandbakk et al., 2011; Moxnes et al., 2013, 2014; Swarén and Eriksson, 2017), but the accuracy of the method has not been thoroughly addressed. The current study addresses the accuracy obtained when applying the power balance principle to GNSS measurements during a roller skiing test race on a World Cup-like ski course. This was achieved using a Monte Carlo simulation sampling from the distributions of the most relevant sources of error (air drag, rolling resistance, variations in wind velocity), which enabled quantification of the method's accuracy throughout the course. Furthermore, the current study is the first to present continuous measurements of propulsive power throughout a test race in distance skiing (Sandbakk et al., 2011; Swarén and Eriksson, 2017). Our findings show that the error in the propulsive power estimates increases with skiing speed, while the propulsive power generated by the athletes decreases approximately linearly as speed increases. They also show that a substantial part of the variability of *P*_prop_ cannot be explained by skiing speed, accelerations, or measurement errors (Figure 5A). Explanations for this variability is most likely the complex course topography, which consisted of both relatively long uphill sections (i.e., from 2,000 to 2,500 m from the start, Figure 4D) and shorter uphill sections where the athletes had recovered during a long downhill section (3,600–3,730 m, Figure 4D). The duration of the longest uphill segment was typically ~120 s, while the short uphill from 3,600 to 3,730 m after start was completed in slightly more than 20 s. During all-out running or cycling tests with durations of 120 and 20 s, the anaerobic energy contributions are approximately 37 and 82%, respectively (Gastin, 2001). Therefore, it is a reasonable assumption that during the longer uphill sections *P*_prop_ is mainly limited by the athletes' VO_2, max_, but this restriction does not apply to uphill sections with short durations, at least if the athletes are in a partially recovered state at the beginning of the uphill. This is in agreement with the observations in the current study, where *P*_prop_ appeared to converge to ~4 W·kg^−1^ in the longest uphill while being almost 8 W·kg^−1^ in the short uphill at 3,600–3,730 m after start.

The observation that propulsive power is higher at low skiing speeds is in agreement with the notion that skiers focus their effort on the uphill sections, where the external resistance is increased due to a substantial component of gravity along the skiing surface. Uphill terrain is also known to be the terrain that is the major determinant of overall performance during time trials (Andersson et al., 2010; Sandbakk et al., 2011, 2016b; Bolger et al., 2015). This is consistent with conclusions in both cycling and cross-country skiing suggesting that athletes should increase their work rate in course segments where the external resistance is increased (Swain, 1997; Atkinson et al., 2007; Sundström et al., 2013). The rationale for this is that a decline in speed over a given distance is not compensated by an equivalent increase in speed over the same distance. This implies that athletes should to some extent aim at minimizing variations in speed by varying the propulsive power.

The peak power outputs measured in the current study are substantially below the values reported by Swarén and Eriksson (2017), who reported peak power outputs of 16 W·kg^−1^ for a male skier during a classical style sprint race. Because the athlete analyzed by Swarén and Eriksson (2017) was a high-level skier (qualified for the final in a Continental Cup race), it would be fair to compare that skier to the best ranked skier in the current study. The best ranked skier in the current study had <15 FIS-points at the time of the data collection, and a peak power output of 8.6 W·kg^−1^, which is still substantially below the findings of Swarén and Eriksson (2017). In contrast, our findings show a larger power output than the segment average power of 5.76 W·kg^−1^ found by Sandbakk et al. (2011) during a sprint skating race. However, this was the average power output over relatively long uphill section (duration of ~1 min) and is therefore difficult to compare directly.

The relationship between propulsive power and metabolic power during ski skating is non-trivial (Sandbakk et al., 2012; Andersson et al., 2017); therefore we cannot deduce directly from our measurements how the metabolic energy demand depends upon skiing speed. However, if we use the measurement of gross efficiency at an 8% incline and a high work rate (η_Gross_ = 16.4 %) from Sandbakk et al. (2012) as a basis, our findings suggests a typical metabolic power of about 38 W·kg^−1^ at skiing speeds of 2 m·s^−1^, which corresponds to an oxygen demand of about 108 mL·kg^−1^·min^−1^ (Péronnet and Massicotte, 1991). For the peak power output measured, the corresponding oxygen demand would be 147 mL·kg^−1^·min^−1^. Assuming a $\dot{\text{V}}$O_2_,_peak_ for this level of skiers of 75 mL·kg^−1^·min^−1^, this corresponds to a typical oxygen demand of about 144% of peak aerobic power at 2 m·s^−1^, and a peak oxygen demand of about 196% of peak aerobic power, and must therefore elicit substantial use of anaerobic energy pathways. This is in agreement with previous studies in both sprint skiing (Sandbakk et al., 2011) and distance skiing (Norman and Komi, 1987; Karlsson et al., 2018). It is interesting to note that the peak metabolic energy requirements in sprint skiing and distance skiing appear to be relatively similar, even though the competition duration is substantially different (~2–4 min for sprint skiing and >30 min for a 15 km race). This might partly explain why cross-country skiing requires a relatively small degree of specialization to each discipline, allowing individual athletes to be world-class over distances ranging from ~1.3 to 50 km.

### Methodological considerations

The method's applicability to discriminate between *P*_prop_ and *F*_prop_ generated by high-level athletes depends on whether the measurement error is smaller than typical inter-athlete and intra-athlete differences observed throughout a race. Our findings show that the proposed method was not sufficiently accurate to discriminate between the inter-athlete or intra-athlete differences observed in this group of high-level skiers. The sources of these errors are distributed almost evenly between air drag (0.034 or 0.016 N·kg^−1^ with measured wind and zero-wind, respectively) and rolling resistance (0.023 N·kg^−1^). Because of varying surface-properties (i.e., asphalt quality) and the significant effect of temperature on the rolling resistance (Ainegren et al., 2008), it is challenging to obtain substantially more accurate measurements of rolling resistance. However, changes in rolling resistance caused by changes in asphalt quality or temperature will to some extent be systematic effects, and will therefore partially cancel when comparing intra- or inter-athlete differences during one experiment. Hence, the sensitivity-criterion used in the current study is appropriate when comparing results from different experiments, but might be too conservative for differences observed during a single experiment.

The results of this study clearly indicate the importance of precise measurements of vertical position. Estimates of propulsive power using vertical position measurements from the standalone GNSS receiver were substantially different (RMS deviation 31% of mean active propulsive power) from the measurements that were mapped onto the dGNSS reference trajectory. Hence, mapping standalone GNSS data on a precisely measured reference trajectory, or using athlete-mounted carrier phase differential GNSS receivers (Gilgien et al., 2014b; Karlsson et al., 2018) is required to calculate meaningful propulsive power measurements. Another solution that might be applicable is to fuse GNSS with IMU or barometric measurements (Skaloud and Limpach, 2003).

Predicting the drag area using only body mass and skiing speed yielded relatively small deviations from the accelerometer-based drag area model, and could therefore be an acceptable solution for many practical applications.

### Limitations

In the current study we used roller skiing as an analog exercise for cross-country skiing on snow. Furthermore, a test race was used rather than an official ski race. Hence, the current study has lower external validity than studies performed on snow (Sandbakk et al., 2011; Swarén and Eriksson, 2017), but has higher internal validity because rolling resistance could be measured more accurately than ski/snow friction, and was less likely to change substantially during the test race.

Another challenge when applying the power balance principle is to accurately calculate air drag, because the drag area and wind speed are difficult to measure continuously along the course. As indicated in Figure 2, the drag coefficient of a cross-country skier depends on the Reynolds number. However, measurements are scarce and inconsistent, particularly at Reynolds numbers <2·10^5^. In the current study we created a model for how the drag coefficient changes with Reynolds number based on measurements on a brass cylinder (Achenbach, 1968), and scaled it to fit previous measurements of ice skaters at 12 m·s^−1^ (van Ingen Schenau et al., 1982). We chose not to base our model on the measurements in Spring et al. (1988), as their equations did not account for changes in gravitational potential energy that would be caused by a non-level rolling surface. This could lead to substantial errors, particularly at low skiing speeds, even if the inclination of the rolling surface is very small. As an example, a 0.1° incline at 3 m·s^−1^ would result in an error in *C*_D_*A* of ~0.25 m^2^. Hence, wind-tunnel studies investigating how the drag coefficient of a cross-country skier depends upon the Reynolds number would be useful, particularly at conditions relevant for low skiing speeds.

A challenge that was not addressed in the drag coefficient model proposed in current study is that cross-country skiing techniques causes body segments to move with different speeds through the air. This effect is most pronounced for the ski poles, where the pole tip's speed varies from 0 (when in contact with the ground) to an unknown speed substantially higher than the skiing speed. However, as the cross-sectional area of ski poles are relatively small, it is likely that this has only a minor effect on the total drag area.

Wind velocity was not measured continuously along the course but was estimated by a wind field based on the hourly average of two nearby meteorological stations, and the assumption of a Rayleigh distribution. The Monte Carlo approach simulated a large number (2,500) of different wind conditions and returned the expected value from all the simulated conditions, which should improve the calculations with respect to the assumption of a constant wind field. Nonetheless, it is obvious that the instantaneous wind velocity is strongly affected by gusts and the proximate surroundings of the course, and our calculations are therefore susceptible to such errors. The errors should however be within the uncertainty bounds specified by the Monte Carlo simulation.

The point mass assumption used in the current study neglects the work required to move body segments with respect to the athlete's center of mass, and therefore does not represent the total mechanical work done by the athlete. It is likely that the total mechanical work substantially exceeds the work required to move the center of mass alone. However, calculation of total mechanical work requires measurements of both the moments of force in all joints and the body segment kinematics (Aleshinsky, 1986; van Ingen Schenau and Cavanagh, 1990). Such measurements are currently not possible, at least in a field situation. Furthermore, even if the total mechanical work could be measured, there is no theoretically valid method to calculate the metabolic energy requirements based on kinetics and kinematics alone. Nevertheless, this should not discourage the use of statistical models linking propulsive power or total mechanical power to metabolic power under the assumption that the model is proven accurate for the problem being studied.

Two additional sources of error that have not been assessed in this study are that the skis do not move the exact same distance as the center of mass (due to the ski skating technique) and the fact that a small fraction of the surface normal force is exerted through the poles (estimated to 3–5% of body weight Millet et al., 1998). These effects have been assessed in other studies (Losnegard et al., 2012; Sandbakk et al., 2012) and are considered to be only of minor consequence.

### Prospects

Studies in other winter sports where equipment-snow/ice friction and air drag are the main opposing forces have shown that the derivations of power, energy/work and propulsive force from athletes using position data are powerful approaches to studying the underlying mechanisms of performance (van Ingen Schenau et al., 1982; Supej, 2008; Gilgien et al., 2014a, 2016, 2018; Kröll et al., 2016). In endurance sports such as cross-country skiing, combining measurements of propulsive power with a model for skiing efficiency is a natural extension of the current study, and would improve our understanding of the physiological requirements of cross-country skiing (Sandbakk et al., 2011; Karlsson et al., 2018). Furthermore, simultaneous measurements of oxygen consumption would allow assessments of the balance between aerobic and anaerobic energy pathways at (or close to) the limit of human endurance racing performance (Andersson et al., 2017). Such measurements could also be used to improve numerical simulations of cross-country skiing performance (Moxnes et al., 2013, 2014), and to explore the effect of different pacing strategies (Swain, 1997; Sundström et al., 2013; Karlsson et al., 2018).

Although the Monte Carlo simulations used in the current study provides some insight into the method's validity and limitations, a validation against a gold standard has not been performed. Future studies could investigate the methods accuracy using ski poles and roller skis instrumented with force transducers.

### Conclusion

During a 13.5 km roller skiing test race on a course similar to a cross-country skiing World Cup competition course, elite cross-country skiers generated a propulsive power output that declined approximately linearly with skiing speed, starting from 6.2 W·kg^−1^ at the lowest measured speeds of 2.0 m·s^−1^ (occurring at inclinations > ~ 10°). At skiing speeds close to 9 m·s^−1^ and inclinations < ~ −2° the skiers transitioned to the tucked position where no propulsive power was generated. Furthermore, the results of this study clearly indicate the importance of precise measurements of vertical position, and shows that standalone GNSS receivers are not sufficiently accurate to be used for propulsive power calculations unless the measurements are mapped on a precisely measured reference trajectory, or replaced by carrier phase differential GNSS receivers. In contrast, predictions of drag area using only body mass and skiing speed deviated only slightly from those based on accelerometer data and should be acceptable for many practical applications. However, none of the methods presented in the current study were sufficiently accurate to discriminate between the instantaneous differences in propulsive force in this group of high-level athletes.

## Author contributions

Conception and design: ØG, TL, MG, DD, and AM-S. Data collection and data analysis: ØG and MG. Manuscript draft of Introduction: ØG and MG. Manuscript draft of Methods, Results, and Discussion: ØG. All authors contributed to manuscript revision, and read and approved the submitted version.

### Conflict of interest statement

The authors declare that the research was conducted in the absence of any commercial or financial relationships that could be construed as a potential conflict of interest.
